# Design and Implementation of a Sun Tracker with a Dual-Axis Single Motor for an Optical Sensor-Based Photovoltaic System

**DOI:** 10.3390/s130303157

**Published:** 2013-03-06

**Authors:** Jing-Min Wang, Chia-Liang Lu

**Affiliations:** Department of Electrical Engineering, St. John's University, 499, Sec. 4, Tam King Road, Tamsui District, New Taipei City 25135, Taiwan; E-Mail: paul@mail.sju.edu.tw

**Keywords:** dual-axis Sun tracker, photovoltaic panel, feedback control theory, light dependent resistor, stand-alone PV inverter, energy gain

## Abstract

The dual threats of energy depletion and global warming place the development of methods for harnessing renewable energy resources at the center of public interest. Solar energy is one of the most promising renewable energy resources. Sun trackers can substantially improve the electricity production of a photovoltaic (PV) system. This paper proposes a novel design of a dual-axis solar tracking PV system which utilizes the feedback control theory along with a four-quadrant light dependent resistor (LDR) sensor and simple electronic circuits to provide robust system performance. The proposed system uses a unique dual-axis AC motor and a stand-alone PV inverter to accomplish solar tracking. The control implementation is a technical innovation that is a simple and effective design. In addition, a scaled-down laboratory prototype is constructed to verify the feasibility of the scheme. The effectiveness of the Sun tracker is confirmed experimentally. To conclude, the results of this study may serve as valuable references for future solar energy applications.

## 1. Introduction

With the rapid increase in population and economic development, the problems of the energy crisis and global warming effects are today a cause for increasing concern. The utilization of renewable energy resources is the key solution to these problems. Solar energy is one of the primary sources of clean, abundant and inexhaustible energy, that not only provides alternative energy resources, but also improves environmental pollution.

The most immediate and technologically attractive use of solar energy is through photovoltaic conversion. The physics of the PV cell (also called solar cell) is very similar to the classical p-n junction diode. The PV cell converts the sunlight directly into direct current (DC) electricity by the photovoltaic effect [1,2]. A PV panel or module is a packaged interconnected assembly of PV cells. In order to maximize the power output from the PV panels, one needs to keep the panels in an optimum position perpendicular to the solar radiation during the day. As such, it is necessary to have it equipped with a Sun tracker. Compared to a fixed panel, a mobile PV panel driven by a Sun tracker may boost consistently the energy gain of the PV panel.

Solar tracking is the most appropriate technology to enhance the electricity production of a PV system. To achieve a high degree of tracking accuracy, several approaches have been widely investigated. Generally, they can be classified as either open-loop tracking types based on solar movement mathematical models or closed-loop tracking types using sensor-based feedback controllers [3–5]. In the open-loop tracking approach, a tracking formula or control algorithm is used. Referring to the literature [6–10], the azimuth and the elevation angles of the Sun were determined by solar movement models or algorithms at the given date, time and geographical information. The control algorithms were executed in a microprocessor controller [11,12]. In the closed-loop tracking approach, various active sensor devices, such as charge couple devices (CCDs) [13–15] or light dependent resistors (LDRs) [12,16–19] were utilized to sense the Sun's position and a feedback error signal was then generated to the control system to continuously receive the maximum solar radiation on the PV panel. This paper proposes an empirical research approach on this issue.

Solar tracking approaches can be implemented by using single-axis schemes [12,19–21], and dual-axis structures for higher accuracy systems [16–18,22–27]. In general, the single-axis tracker with one degree of freedom follows the Sun's movement from the east to west during a day while a dual-axis tracker also follows the elevation angle of the Sun. In recent years, there has been a growing volume of research concerned with dual-axis solar tracking systems. However, in the existing research, most of them used two stepper motors [22,23] or two DC motors [16,17,24,25] to perform dual-axis solar tracking. With two tracking motors designs, two motors were mounted on perpendicular axes, and even aligned them in certain directions. In some cases, both motors could not move at the same time [5]. Furthermore, such systems always involve complex tracking strategies using microprocessor chips as a control platform. In this work, employing a dual-axis with only single tracking motor, an attempt has been made to develop and implement a simple and efficient control scheme. The two axes of the Sun tracker were allowed to move simultaneously within their respective ranges. Utilizing conventional electronic circuits, no programming or computer interface was needed. Moreover, the proposed system used a stand-alone PV inverter to drive motor and provide power supply. The system was self-contained and autonomous. Experiment results have demonstrated the feasibility of the tracking PV system and verified the advantages of the proposed control implementation.

The remainder of the article is organized in the following manner: Section 2 describes the tracking strategies of the developed closed-loop solar tracking system in which a sensor-based feedback controller is used. The detailed architecture of the Sun tracker hardware is proposed in Section 3. In Section 4, a scaled-down laboratory prototype is built and tested. Finally, the main conclusions of this work are drawn in Section 5.

## 2. Developed Closed-Loop Solar Tracking System

**Figure 1.**
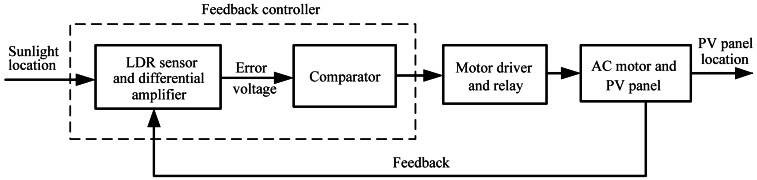
Block diagram of the solar tracking system.

The block diagram of the developed closed-loop solar tracking system is illustrated in Figure 1, describing the composition and interconnection of the system. For the closed-loop tracking approach, the solar tracking problem is how to cause the PV panel location (output) to follow the sunlight location (input) as closely as possible. The sensor-based feedback controller consists of the LDR sensor, differential amplifier, and comparator. In the tracking operation, the LDR sensor measures the sunlight intensity as a reference input signal. The unbalance in voltages generated by the LDR sensor is amplified and then generates a feedback error voltage. The error voltage is proportional to the difference between the sunlight location and the PV panel location. At this time the comparator compares the error voltage with a specified threshold (tolerance). If the comparator output goes high state, the motor driver and a relay are activated so as to rotate the dual-axis (azimuth and elevation) tracking motor and bring the PV panel to face the Sun. Accordingly, the feedback controller performs the vital functions: PV panel and sunlight are constantly monitored and send a differential control signal to drive the PV panel until the error voltage is less than a pre-specified threshold value.

**Figure 2.**
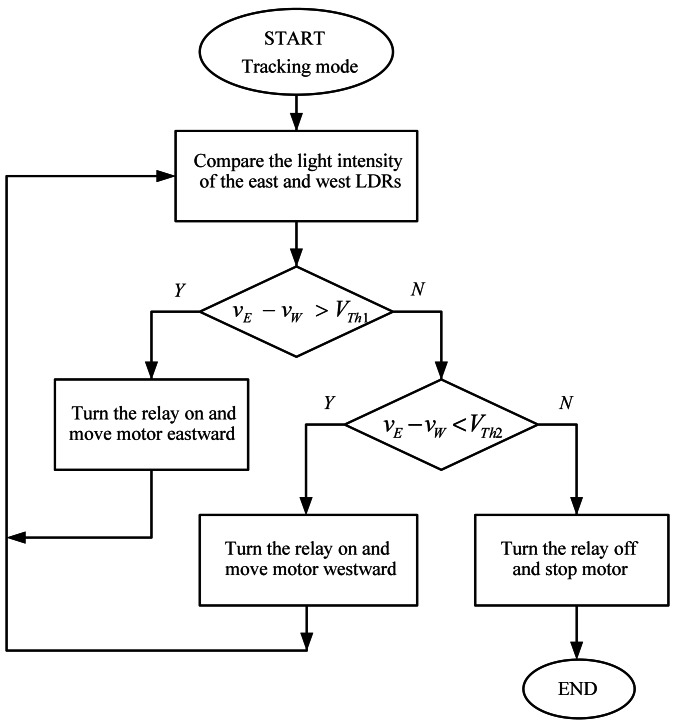
Flowchart of tracking algorithm for azimuth control.

**Figure 3.**
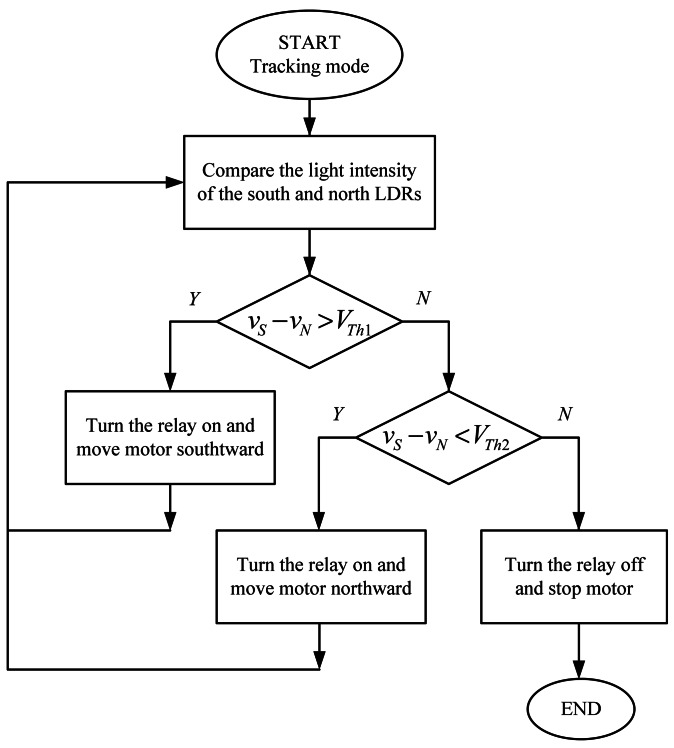
Flowchart of tracking algorithm for elevation control.

The system tracks the Sun autonomously in azimuth and elevation angles. The whole working algorithms are summed up in the flowcharts shown in Figures 2 and 3. The sunlight intensity from four different directions is measured by the LDR-based sensing circuit. The voltages *v_E_*, *v_W_*, *v_S_* and *v_N_* are defined as the sensing voltages produced by the east, west, south, and north LDRs respectively. In an attempt to draw maximum power from the PV panel, the azimuth and elevation tracking processes can simultaneously proceed until the PV panel is aligned orthogonally to the sunlight. The tracker installation is not restricted to the geographical location.

## 3. Sun Tracker Hardware Design

**Figure 4.**
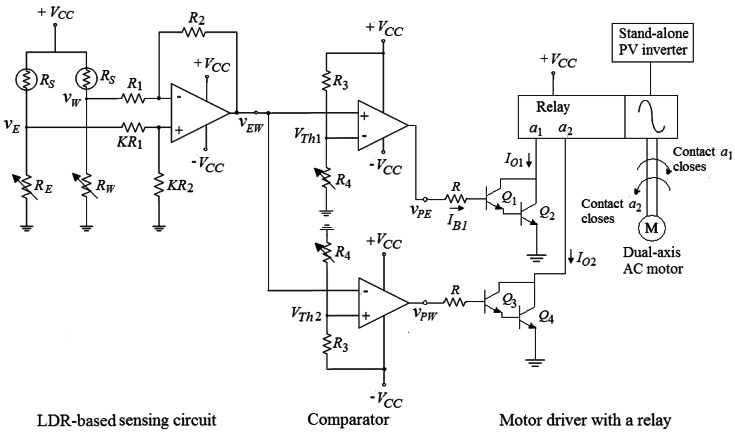
Complete control circuit diagram of the Sun tracker for azimuth tracking.

Figure 4 presents one of the hardware circuits of the proposed Sun tracker for azimuth tracking. The entire system has two hardware circuits with both of azimuth direction and elevation direction to drive the dual-axis AC motor. The developed Sun tracker is comprised of three modules, which are the LDR-based sensing circuit, comparator and a motor driver with a relay.

### 3.1. LDR-Based Sensing Circuit

To track the sunlight, it is necessary to sense the position of the Sun and for that an electro-optical sensor is needed. The proposed Sun tracker uses the electro-optical sensor for self-calibration. A LDR or photoresistor is a variable resistor whose electrical resistance depends on the intensity of the light falling on it. The LDR resistance decreases with incident light intensity increasing. As seen in the first part of Figure 4, the LDR sensor is a part of the voltage divider circuit in order to give an output voltage.

#### 3.1.1. Solar Sensing Device

**Figure 5.**
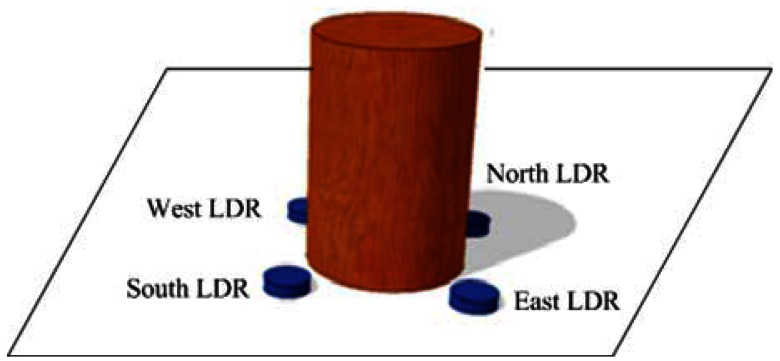
Solar sensing device with a four-quadrant LDR sensor.

The paper creates a Sun tracker using LDRs with a cylindrical shade as a Sun sensor. Figure 5 shows the designed solar sensing device, which comprises a four-quadrant LDR sensor and a cylinder mounted on a wood-block. The solar sensing device is attached to the PV panel. The East/West LDR and the South/North LDR are respectively used in the detection of azimuth motion and elevation motion of the PV panel. The design of the light sensor is based on the use of the shadow. If the PV panel is not perpendicular to the sunlight, the shadow of the cylinder will cover one or two LDRs and this causes different light intensity to be received by the sensing device.

#### 3.1.2. Creating Feedback Error Voltage

The simple electronic circuit for creating error voltage is presented in the first part of Figure 4. It can be seen that the output voltage of the voltage divider will be lower when the corresponding LDR is shadowed. If one sensor is lighted and the other is shadowed, the differential amplifier amplifies the difference voltage between them. The feedback error voltage can be expressed as:

(1) $$v_{EW}=-\frac{R_{2}}{R_{1}}v_{W}+(1+\frac{R_{2}}{R_{1}})\frac{KR_{2}}{KR_{1}+KR_{2}}v_{E}$$

which can be rearranged as follows:

(2) $$v_{EW}=\frac{R_{2}}{R_{1}}\;(v_{E}-v_{W})$$

If the west LDR is shaded, *v_E_* > *v_W_* and *v_EW_* > 0.

### 3.2. Comparator

The main function of the comparator is to act as a switch to turn on the relay and rotate the motor. A comparator is essentially an operational amplifier (op-amp) operated in an open-loop configuration, which converts a time-varying analog signal into a binary output. As depicted in the second part of Figure 4, the comparator is designed to compare the error voltage with two threshold values. The threshold value is defined as the input voltage at which the output changes states. As shown in Figure 4, there are two threshold values which are given as:

(3) $$V_{Th1}=\frac{R_{4}}{R_{3}+R_{4}}V_{CC}$$

(4) $$V_{Th2}=\frac{R_{4}}{R_{3}+R_{4}}(-V_{CC})$$

The output of the comparator is a high saturated state *V_H_* or a low saturated state *V_L_*. The saturated output voltages *V_H_* and *V_L_* may be closed to the supply voltages +*V_CC_* and −*V_CC_*, respectively. The comparator outputs are then expressed as follows:

(5) $$\begin{array}{ccc} v_{PE} & =V_{H} & \text{for}\;v_{EW}>V_{Th1}\\ & =V_{L} & \text{for}\;v_{EW}<V_{Th1} \end{array}$$

(6) $$\begin{array}{ccc} v_{PW} & =V_{L} & \text{for}\;v_{EW}>V_{Th2}\\ & =V_{H} & \text{for}\;v_{EW}<V_{Th2} \end{array}$$

**Figure 6.**
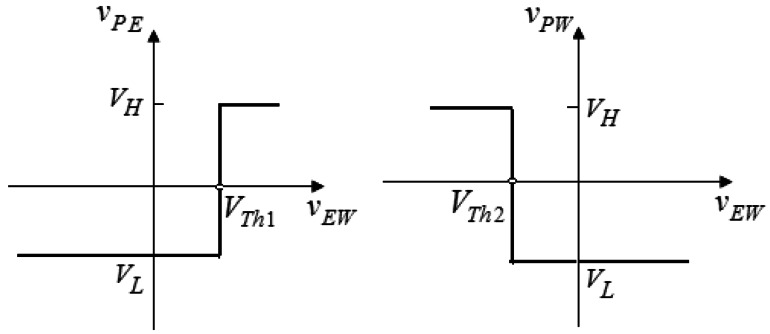
Voltage transfer characteristics of the open-loop comparator.

The voltage transfer characteristics of the comparator with ideal op-amps are shown in Figure 6. It is noted that the sensitivity for the tracking system is dominated by the threshold values, which are adjusted by the variable resistor *R*_4_ according to the tracking accuracy. As *R*_4_ decreases, the tracking accuracy increases. However, the system tracking response will become increasingly oscillatory.

### 3.3. Motor Driver with a Relay

As seen in the last part of Figure 4, it is observed that the designed motor driver with a relay consists of two Darlington pairs that provide increased current gain and actuate the relay. If the west LDR is shaded, a feedback error voltage *v_EW_* is generated. When *v_EW_* > *V_Th_*_1_ > *V_Th_*_2_, the comparator outputs *v_PE_* and *v_PW_* go high and low saturated voltages respectively. The transistors *Q*_1_ and *Q*_2_ will therefore conduct and *Q*_3_ and *Q*_4_ are in the cutoff state. Referring to Figure 4, transistors *Q*_1_ and *Q*_2_ operate in the forward-active mode, and the input current or the base current of *Q*_1_ is:

(7) $$I_{B1}=\frac{V_{H}-V_{BE1}-V_{BE2}}{R}$$

where *V_BE_* is the forward-biased base-emitter voltage of the bipolar transistor. Therefore, the output current can be written as:

(8) $$I_{O1}=I_{C1}+I_{C2}=\beta_{1}I_{B1}+\beta_{2}(1+\beta_{1})I_{B1}=[\beta_{1}(1+\beta_{2})+\beta_{2}]I_{B1}≅\beta_{1}\beta_{2}I_{B1}$$

The parameters *β*_1_ and *β*_2_ are the common-emitter current gain of the bipolar transistors.

The relay is activated by the output current and normally-open contact *a*_1_ closes. In this case, the tracking motor rotates clockwise in the azimuth direction and thus the PV panel moves eastward to face the Sun. More specifically, the Sun tracker attempts to adjust the PV panel such that all the voltages produced by LDRs are nearly equal and balance. As a result, the PV panel is almost perpendicular to the sunlight and has a high energy generation.

### 3.4. Stand-Alone PV Power System

**Figure 7.**
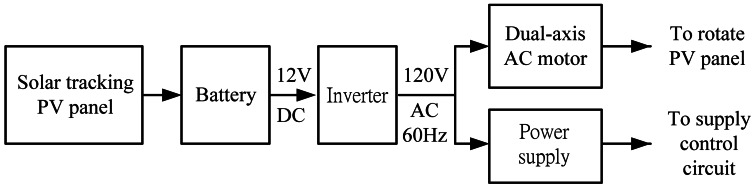
Stand-alone PV power system.

A stand-alone or off-grid PV power system is designed to charge DC power generated by PV panel into battery and convert the DC electricity into AC source for AC appliances. The stand-alone power system is depicted in Figure 7. The inverter changes 12 V DC input voltage to 120 V AC output voltage used to power the AC motor and provide the power supply. The motor rotates the PV panel, and the power supply provides a DC source to the entire circuit. In other words, the developed system has an autonomous energy supply.

## 4. Experiment Validation

To verify the performance of the Sun tracker and to illustrate the feasibility of the control scheme, a laboratory prototype of the solar tracking system was built and tested. This work was done in New Taipei City, Taiwan on 16 May 2012. It was partly cloudy day. The latitude of Taiwan is 23.5 degrees north, so that the fixed panel was faced the south at a tilt angle of 23.5 degrees with maximum average solar radiation.

### 4.1. Experiment Tests

**Figure 8.**
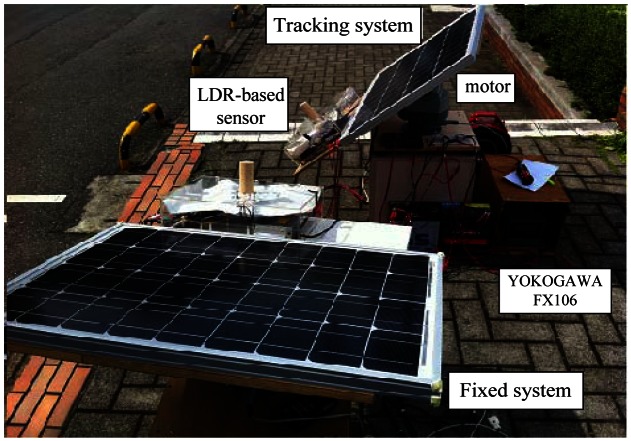
Actual photograph of the experimental prototype.

Figure 8 shows the experiment tests on the polycrystalline PV panel with the Sun tracker and with a fixed angle. The physical dimensions (*L × W × D*) of the PV panel were 835 mm × 518 mm × 30 mm. The chosen unique motor for this work was dual-axis 117V AC motor (LILIN PIH-303) with a speed of 6°/s in pan and 3°/s in tilt. It had two degrees of freedom to move the panel in the azimuth angle and the altitude angle simultaneously. The work employed paperless recorder (YOKOGAWA FX106) to display the real-time measurement of the voltage and current generated by PV panel.

The Earth rotates 15 degrees with respect to its axis per hour, and the Sun moves slowly [26,28]. To minimize power consumption, the tracking motor was not operated continuously. The developed system thus adopted a time schedule algorithm to follow the Sun about every half an hour by varying the resistor *R*_4_.

### 4.2. Experiment Results

**Figure 9.**
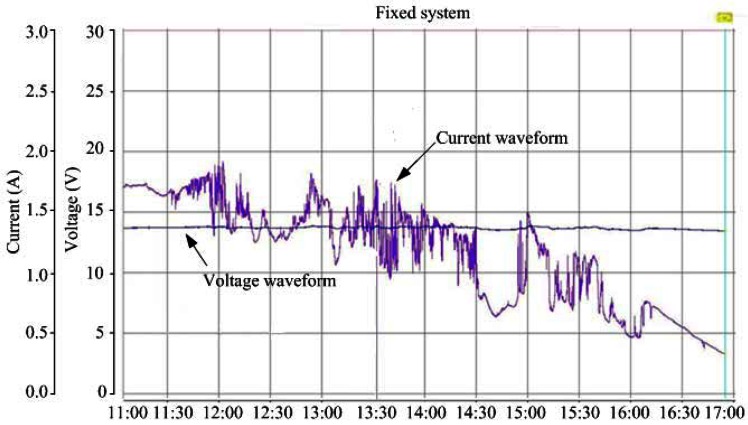
Measured time functions of output voltage and current for fixed PV system.

**Figure 10.**
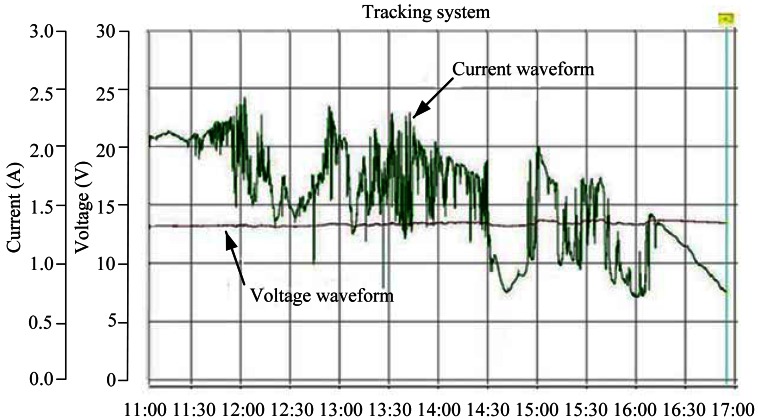
Measured time functions of output voltage and current for solar tracking system.

**Figure 11.**
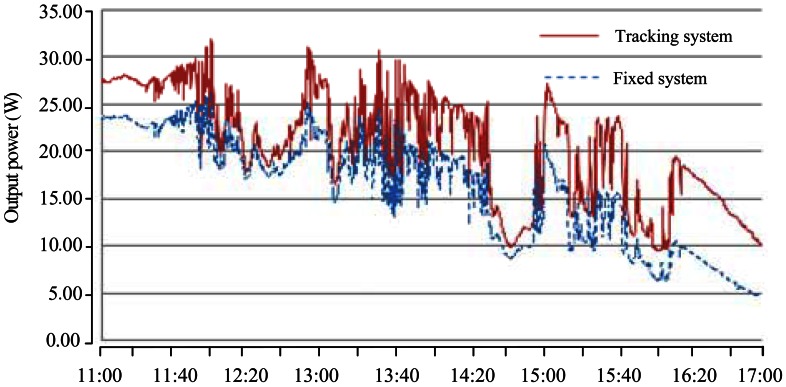
Comparison of the generating powers for the tracking and fixed systems.

The measurement results of generating voltage and current waveforms for the fixed and tracking PV systems are shown in Figures 9 and 10, respectively. In order to give a clear explanation on the energy productivity, the generating instantaneous powers of the both systems were further calculated. Figure 11 shows the test results with promising outcome.

### 4.3. Performance Analysis

As shown in Figure 11, the performance of the tracking system was significantly less at noon. It could be reasoned that both of the PV panels almost faced the same direction, but a slight difference of angle. The increase in power was focused in the afternoon. The generating electrical energies for the fixed and tracking systems were 95.52 W-h and 123.06 W-h, respectively. Therefore, an extra yield of (123.06−95.52)/95.52 = 0.2883 or 28.83% energy generation was obtained. The increased solar energy was consistent with the existing literature [18,23,27].

Energy gain is the most important factor in evaluating a tracking system. The energy gain means how much the tracking system increases the energy generated compared with a fixed system. However, to calculate the net energy gain of the tracking system, energy consumption of the rotating motor should be considered. The motor energy consumption was 0.5 W-h. The energy gain of the tracking system was (123.06−95.52−0.5)/95.52 = 0.2831 or 28.31%. The partly cloudy day would affect the energy generation. Moreover, if the tracking frequency was adjusted reasonably according to actual tracking condition, the improved electricity production can be further enhanced.

## 5. Conclusions

The paper has presented a novel and a simple control implementation of a Sun tracker that employed a single dual-axis AC motor to follow the Sun and used a stand-alone PV inverter to power the entire system. The proposed one-motor design was simple and self-contained, and did not require programming and a computer interface. A laboratory prototype has been successfully built and tested to verify the effectiveness of the control implementation. Experiment results indicated that the developed system increased the energy gain up to 28.31% for a partly cloudy day.

The proposed methodology is an innovation so far. It achieves the following attractive features: (1) a simple and cost-effective control implementation, (2) a stand-alone PV inverter to power the entire system, (3) ability to move the two axes simultaneously within their respective ranges, (4) ability to adjust the tracking accuracy, and (5) applicable to moving platforms with the Sun tracker. The empirical findings lead us to believe that the research work may provide some contributions to the development of solar energy applications.
